# Differential performance regarding the relationship of C3-epi-25(OH)D3 levels and %C3-epi-25(OH)D3 with common pediatric diseases: a case control study

**DOI:** 10.1186/s12887-024-05072-8

**Published:** 2024-09-13

**Authors:** Tao Yang, Xiaohong Chen, Miyan Wang, Shaohua Xu, Dong Hu, Jie Tang, Yuwei Yang

**Affiliations:** 1 Department of Laboratory Medicine, Santai Country People’s Hospital, Mianyang, 621100 P.R. China; 2 Department of Laboratory Medicine, School of Medicine, Mianyang Central Hospital, University of Electronic Science and Technology of China, Mianyang, 621000 P.R. China; 3 Department of Paediatric Surgery, School of Medicine, Mianyang Central Hospital, University of Electronic Science and Technology of China, Mianyang, 621000 P.R. China

**Keywords:** C3-epimer, 25(OH)D3, Influence factor, Correlation, Paediatric diseases

## Abstract

**Background:**

Recently, the C3-epimer of 25-hydroxyvitamin D [C3-epi-25(OH)D] has become a topic of interest among 25-hydroxyvitamin D [25(OH)D] metabolites. Although it can lead to an overestimation of vitamin D storage, its relationship with disease occurrence remains controversial, possibly related to the great extent of tracking of 25(OH)D by C3-epi-25(OH)D over time. This study aimed to investigate the differential performance of C3-epi-25(OH)D3 and its percentage [%C3-epi-25(OH)D3] with respect to 20 common paediatric diseases.

**Methods:**

This study involved 805 healthy children and adolescents and 2962 patients with common paediatric diseases. We investigated sex, age, and seasonal differences in C3-epi-25(OH)D3 and %C3-epi-25(OH)D3 levels; their variations on 20 common paediatric diseases; and their degree of correlation with 25(OH)D3 levels and various diseases.

**Results:**

Among the healthy underage participants, C3-epi-25(OH)D3 and %C3-epi-25(OH)D3 changed similarly, with no sex differences. Moreover, their levels were higher in the infant period than in the other periods (*t* = 5.329–5.833, *t* = 4.640–5.711, all *Padj* < 0.001), and in spring and summer than in autumn and winter (*t* = 3.495–6.061, *t* = 3.495–5.658, all *Padj* < 0.01). Under healthy and disease conditions, C3-epi-25(OH)D3 was positively correlated with 25(OH)D3 (*ρ* = 0.318 ~ 0.678, all *P* < 0.017), whereas %C3-epi-25(OH)D3 was not, except in patients with nephrotic syndrome (*ρ*=-0.393, *P* = 0.001). Before and after adjusting for 25(OH)D3, the relationship of C3-epi-25(OH)D3 with the diseases was notably different. However, it was almost consistent for %C3-epi-25(OH)D3. Our results indicated that %C3-epi-25(OH)D3 was associated with short stature, nephrotic syndrome, lymphocytic leukaemia, rickets, paediatric malnutrition, and hypovitaminosis D (*OR* = 0.80 ~ 1.21, all *P* < 0.05).

**Conclusions:**

The %C3-epi-25(OH)D3 can correct the properties of C3-epi-25(OH)D3 to better track 25(OH)D3 and may be more suitable for exploring its pathological relevance. Further detailed studies of each disease should be conducted.

## Background

The level of 25-hydroxyvitamin D [25(OH)D] in vivo is a recognized criterion for determining vitamin D storage and is also recommended by the Institute of Medicine (IOM) [1, 2] and the Endocrine Society (EnS)/Japan Endocrine Society (JES) [3, 4]. However, these criteria are controversial. The IOM and EnS/JES defined vitamin D deficiency as 25(OH)D < 12 ng/mL (30 nmol/L) and < 20 ng/mL (50 nmol/L), respectively. Moreover, the IOM indicates that 25(OH)D of 50 nmol/L is sufficient to ensure bone health in the general population [5].

The physiological roles of vitamin D include the regulation of calcium and phosphate metabolism [6], the regulation of neuromuscular function [7], immune-modulatory function [8], antioxidation [9], and antiproliferative effects [10], among others. Thus, vitamin D deficiency is associated with impaired absorption of calcium and phosphorus, inflammation, immune dysfunction, oxidative stress, and tumorigenesis, resulting in an increased risk of various diseases, such as rickets, osteomalacia, cardiovascular disease, and autoimmune diseases [11, 12].

The level of the C3-epimer of 25(OH)D [C3-epi-25(OH)D] in vivo has also raised concerns based on the nutritional evaluation of vitamin D [11]; that is, there are concerns regarding the correct evaluation of vitamin D storage. C3-epi-25(OH)D, another form of 25(OH)D, is generated mainly from 3β→3α epimerization at the C3 spatial conformation of 25(OH)D by the 3-epimerase enzyme. Owing to its low or no bioactivity, C3-epi-25(OH)D can cause an overestimation of vitamin D storage [13]. However, there are two urgent problems in the measurement of C3-epi-25(OH)D: (1) owing to the similar spectroscopic patterns and identical mass and fragmentation patterns of 25(OH)D and C3-epi-25(OH)D, almost no spectroscopy detection technique can distinguish them; (2) owing to the very low levels in the general population, C3-epi-25(OH)D is very easily identified as signal noise using ordinary mass-spectrography techniques, except when its content is high. In 2006, it was reported that the in vivo concentration of C3-epi-25(OH)D could be determined via high-performance liquid chromatography-tandem mass spectrometry (HPLC-MS/MS) [14]. However, assessments of C3-epi-25(OH)D in vivo have been in progress for less than 20 years and have been conducted in few developed countries [15, 16]. Nevertheless, C3-epi-25(OH)D remains undetectable in many individuals.

C3-epi-25(OH)D has become a topic of interest in the field of vitamin D nutrition, particularly regarding its controversial pathological relevance. Studies have shown that C3-epi-25(OH)D levels increase in various conditions and diseases, such as infants, children, pregnant women, autoimmune diseases, arthritis, diabetes mellitus, Alzheimer’s disease, and thyroid disorders [17–19]. One study revealed that the C3-epi-25(OH)D level tracks the 25(OH)D level to a great extent over time [20]. These findings indicate that the C3-epi-25(OH)D3 level is strongly affected by the 25(OH)D3 level in the peripheral blood. Therefore, most studies have postulated that C3-epi-25(OH)D plays a role in the overestimation of vitamin D storage [21]. However, little is known regarding the relationship between C3-epi-25(OH)D and diseases. Recent genome-wide studies have confirmed that genetic factors can influence various pathways of vitamin D metabolism and that vitamin D metabolism gene polymorphisms can influence the epimerization process itself [22, 23]. Therefore, the level of C3-epi-25(OH)D in peripheral blood not only causes an overestimation of vitamin D storage but also may have pathological significance. Our previous study revealed for the first time that C3-epi-25(OH)D may be a superior marker to 25(OH)D for predicting the severity of chronic kidney disease in patients with rheumatoid arthritis [24]. Therefore, we hypothesized that C3-epi-25(OH)D tracks 25(OH)D to a great extent and may explain the difficulty in determining the pathological value of C3-epi-25(OH)D.

In this regard, we used an HPLC‒MS/MS method to assess C3-epi-25(OH)D3 levels in vivo and adopted the C3-epi-25(OH)D3% [%C3-epi-25(OH)D3] to correct for the influence of 25(OH)D3 levels, then compared the differential performance of C3-epi-25(OH)D3 level and %C3-epi-25(OH)D3 in common paediatric diseases. C3-epi-25(OH)D2 could not be analysed via our detection technique because of its relatively low content.

## Methods

### Participants

**Fig. 1:**
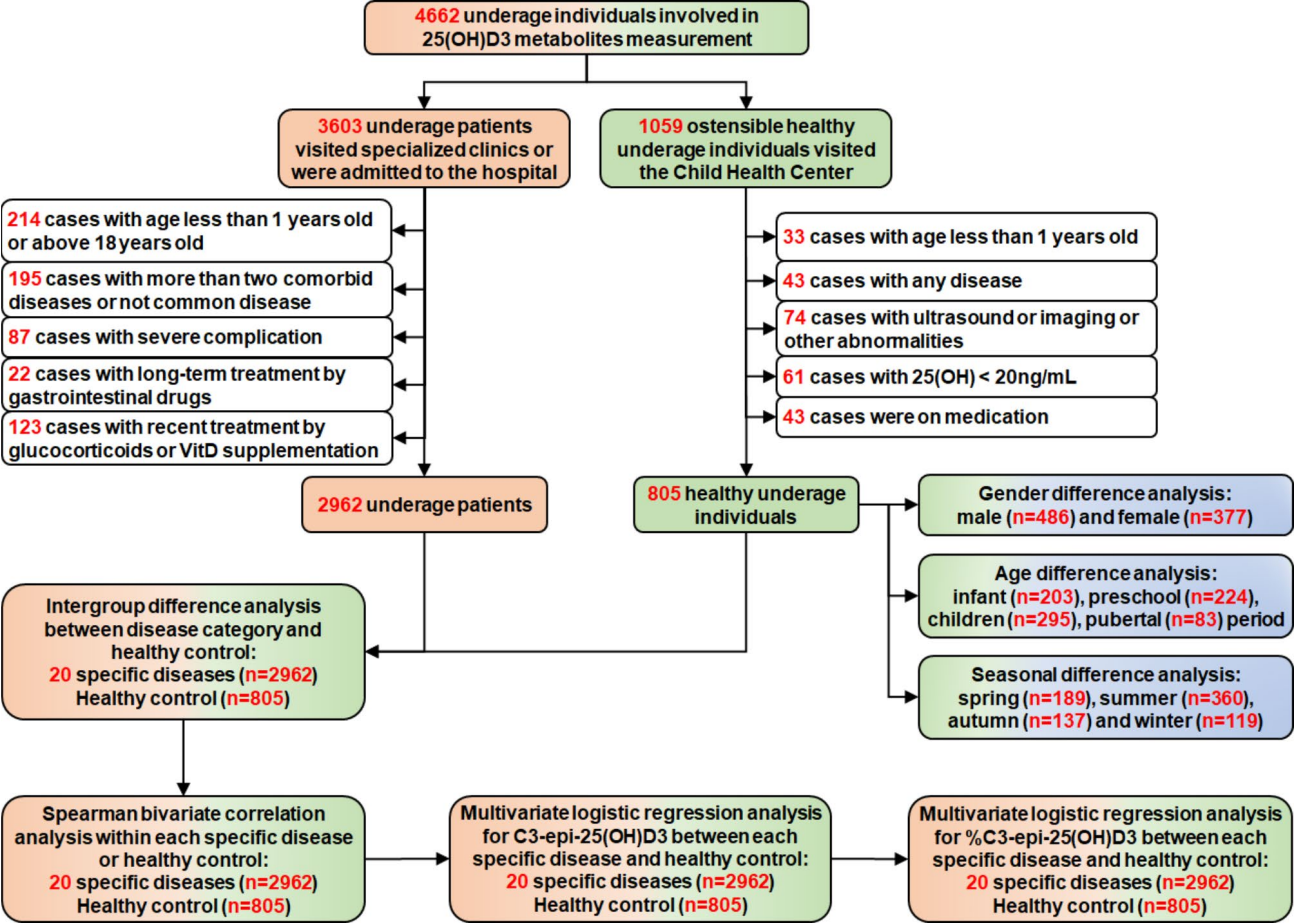
The participant flowchart

From November 2020 to June 2022, 4662 healthy paediatric participants and patients were involved in the determination of 25(OH)D metabolites. According to the inclusion and exclusion criteria, of the 4662 subjects screened for this study, 805 healthy underage participants and 2962 patients with common paediatric diseases were enrolled, including 2076 males and 1691 females aged 1 − 18 years (average age, 7.5 ± 3.7 years). The flowchart of the participants included in the analysis is shown in Fig. 1.

#### Inclusion and exclusion criteria for healthy paediatric controls

The inclusion criteria were as follows: (1) aged above 1 year and less than 18 years; (2) healthy participants who attended the Child Health Center; (3) no visible physical impairment or disability; (4) normal cardiac, pulmonary, hepatic, and renal function and no abnormalities on routine blood and urine examination.

The exclusion criteria were as follows: (1) age less than 1 year and above 18 years; (2) diagnosis of any definite disease or illness after general medical examination; (3) obvious abnormalities in hepatic and renal function tests, complete blood count, and routine urine test; (4) obvious abnormalities on ultrasound or imaging examination (if checked); (5) taking any medication or vitamin D supplementation within 1 month.

#### Inclusion and exclusion criteria for paediatric patients

The inclusion criteria were as follows: (1) age above 1 year and less than 18 years; (2) patients who were followed up at the Child Health Center or the Paediatric Specialist Clinic or prepared for hospital admission; (3) disease diagnosis that strictly adhered to the cluster coding of the WHO International Classification of Diseases [25]; and (4) single disease diagnosis whenever possible, without comorbid diseases or severe complications.

The exclusion criteria were as follows: (1) age less than 1 year and above 18 years, (2) presence of more than two comorbid diseases, (3) presence of severe complications in other tissues or organs, (4) presence of uncommon paediatric diseases, and (5) long-term treatment with gastrointestinal drugs (containing sucralfate or aluminium hydroxide), taking glucocorticoids within 1 week, or taking vitamin D supplementation within 1 month.

### Sample collection and processing

Approximately 3.0 mL of fasting venous blood was collected from all participants in SST-II vacuum tubes (BD, USA) early in the morning. The blood samples were centrifuged at approximately 1500 ×g for 10 min to separate the serum, which was then used for 25(OH)D metabolite determination. When not used for immediate assessment, the serum samples were stored at 2–8℃ for less than 7 days.

On the day of assessment, the serum samples were pretreated as follows. Specifically, 200 µL of the serum sample was mixed with 10 µL of internal standard (a mixture of 25[OH]D3-d6 and C3-epi-25[OH]D3-[2H3]). The mixture was then vortexed with 1.0 ml of tert-butyl methyl ether release agent (CNW, Germany) for 5 min and centrifuged at 12,000 ×g for 5 min. Then, 800 µL of the supernatant was collected and dried using an MD200-1 A Nitrogen Evaporator (Allsheng, China). The blow-dried substance was redissolved in 100 µL of methanol solution containing 0.1% formic acid, vortexed for 2 min, and centrifuged at 12,000 ×g for 5 min. Then, 50 µL of the supernatant was collected to measure the levels of 25(OH)D metabolites.

### Isolation, identification, and measurement of 25(OH)D3 metabolites

The 25(OH)D3 metabolite levels were measured using a lipid-soluble vitamin assay kit (FINDBiotech, Chengdu) on a Jasper™ HPLC-MS/MS (Shimadzu, Japan)/AB SCIEX™ 4500MD triple quadrupole mass spectrometer (ABI, USA). This method has been reported in our previous study [24]. After rigorous performance verification tests were performed, the lower limits of quantitation for 25(OH)D3 and C3-epi-25(OH)D3 were determined to be 0.95 ng/ml and 0.40 ng/ml, respectively; the recoveries of standard addition for three levels of 25(OH)D3 (5.0ng/mL, 30ng/mL, and 70ng/mL) during the five repeated measurements were observed to be within the ranges of 92.0%~114.8%, 88.2%~113.8%, and 90.6%~114.1%, respectively. Furthermore, the monthly coefficient of variation for 25(OH)D3 dual-level quality control (15.19ng/mL and 55.34ng/mL) was calculated during the study period, yielding results of 2.46%~5.95% and 2.39%~5.36%, respectively. The separate monitoring of C3-epi-25(OH)D3 was not conducted due to its highly similar mass spectrometry characteristics to 25(OH)D3. On the basis of the measured results, %C3-epi-25(OH)D3 was defined as the C3-epi-25(OH)D3 concentration divided by the sum of the C3-epi-25(OH)D3 and 25(OH)D3 concentrations.

### Statistical analysis

Data analysis was performed using the SPSS software v22.0 (SPSS, USA) and MedCalc software v20.1 (MedCalc, Belgium). After confirming a nonnormal distribution via a one-sample Kolmogorov‒Smirnov test, the measurement data were described as medians (*P*_*25*_, *P*_*75*_) [*min*, *max*]. The overall differences in sex, age, and season were analysed via the covariance analysis. To correct for the influence of confounding factors, pairwise comparisons were performed using multiple comparisons of covariance analysis with Bonferroni correction, and the significance was determined by the adjusted P value (*Padj*). Spearman correlation analysis was used to investigate the correlation of C3-epi-25(OH)D3 levels and %C3-epi-25(OH)D3 with 25(OH)D3 levels. Multinomial logistic regression was used to investigate the correlations of C3-epi-25(OH)D3 levels and %C3-epi-25(OH)D3 with diseases, and odds ratios (*ORs*) and 95% confidence intervals (*CIs*) were used to assess the degrees of their relationships. A *P* or *Padj* < 0.05 was considered statistically significant except for Spearman’s correlation analysis (*P* < 0.017).

## Results

### Degrees of influence of sex, age, and season on C3-epi-25(OH)D3 generation

**Fig. 2:**
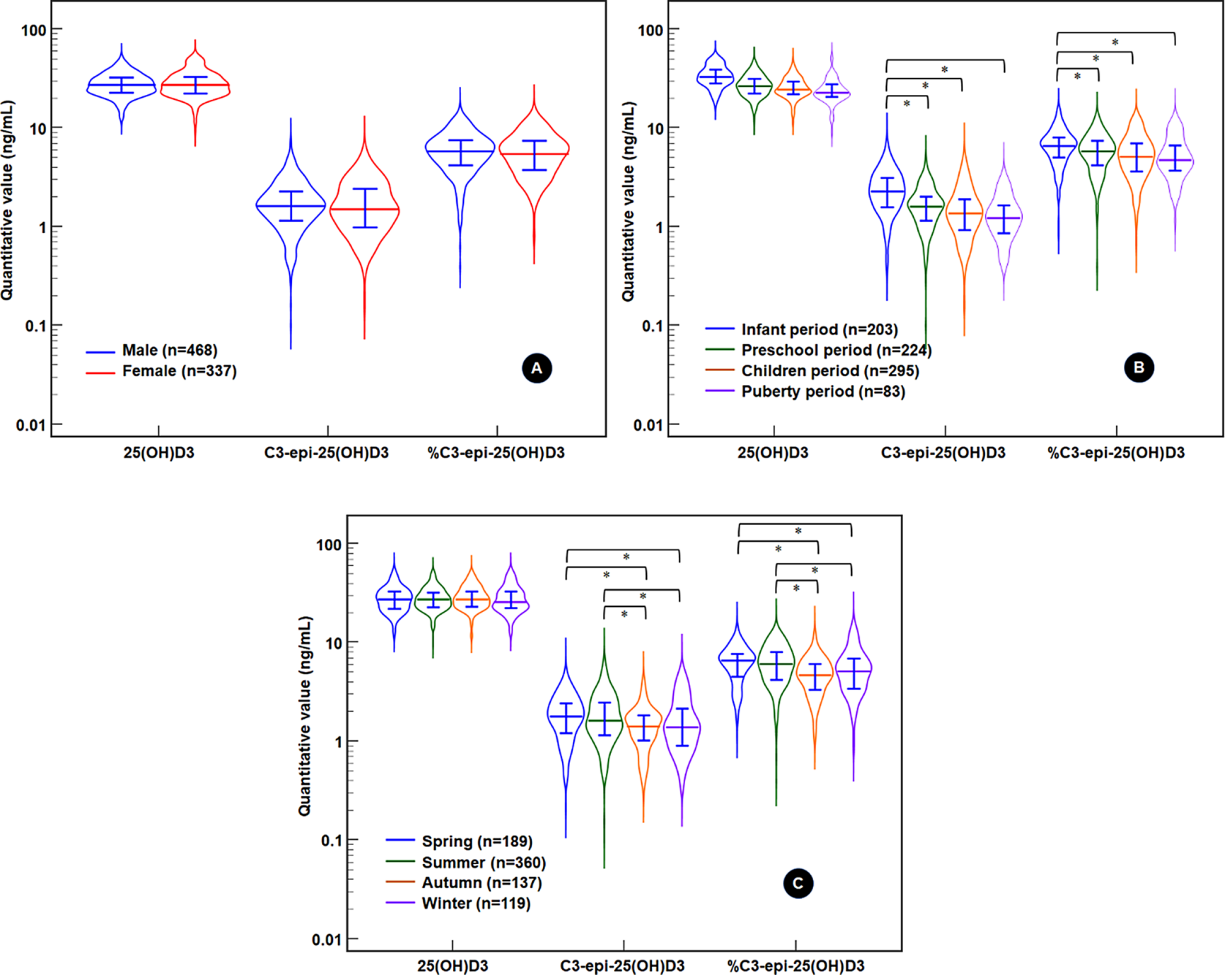
Gender, age, and season differences in C3-epi-25(OH)D3 levels and percentages among 805 healthy underage individuals. *Note* * After the Bonferroni correction, Padj < 0.05. The 25(OH)D3 distribution is only shown without analysis. The violin plot presents a density trace of detailed distributional characteristics. The longer horizontal line represents the median, and the top and bottom horizontal lines of I shape represent P75 and P25, respectively. Both the C3-epi-25(OH)D3 level and percentage are greater in the infant period than in other periods and are greater in spring and summer than in autumn and winter

First, we focused on the degree of influence of age, sex, and season on C3-epi-25(OH)D3 generation in 805 healthy underage individuals. The group settings for sex, age, and season were as follows: male (*n* = 486) and female (*n* = 377); infant period (*n* = 203, 1 to less than 3 years old); preschool period (*n* = 224, 3 to less than 6 years old); child period (*n* = 295, 6 to less than 12 years old); pubertal period (*n* = 83, 12 to less than 18 years old); spring (*n* = 189, Mar. to May); summer (*n* = 360, Jun. to Aug.); autumn (*n* = 137, Sep. to Nov.); and winter (*n* = 119, Dec. to Feb.). To correct for the influence of age, sex, or seasonal factors, we performed covariance analysis for each factor while controlling for the remaining two factors along with 25(OH)D3 levels as confounding factors (shown in Fig. 2). The C3-epi-25(OH)D3 levels and %C3-epi-25(OH)D3 did not differ according to sex (*F* = 1.107 and 2.164, *P* = 0.293 and 0.142) but did differ according to age (*χ*2 = 14.981 and 13.052, both *P* < 0.001) and season (*F* = 13.784 and 12.460, both *P* < 0.001).

Multiple comparisons using Bonferroni correction revealed that the C3-epi-25(OH)D3 levels were higher in the infant period than in the other periods (*t* = 5.329–5.833, all *Padj* < 0.001). They were also higher in spring and summer than in autumn and winter (*t* = 3.495–6.061, all *Padj* < 0.01). Moreover, the %C3-epi-25(OH)D3 was higher in the infant period (*t* = 4.640–5.711, all *Padj* < 0.001) than in the other periods. It was also higher in spring and summer than in autumn and winter (*t* = 3.495–5.658, all *Padj* < 0.01). Our results confirmed the physiological increase in C3-epi-25(OH)D3 levels and %C3-epi-25(OH)D3 during infancy, as well as in spring and summer. Therefore, both can reflect the physiological generation of the 25(OH)D3 C3-epimer.

### C3-epi-25(OH)D3 levels and %C3-epi-25(OH)D3 in common paediatric diseases

A total of 2962 cases of common paediatric diseases were assessed in this study. Moreover, the disease categories (specific diseases) included skeletal system diseases (hypovitaminosis D, short stature, rickets, and growing arthralgia), respiratory system diseases (pneumonia, atopic cough, trachitis/bronchitis, upper respiratory infection, and cough variant asthma), endocrine system diseases (thyroid disorder, growth hormone deficiency, and sexual precocity), neurological system diseases (tic disorder, epilepsy, attention-deficit/hyperactivity disorder, mental retardation/developmental disabilities, and sleep disorders), and other system diseases (nephrotic syndrome, paediatric malnutrition, and lymphocytic leukaemia). Short stature was defined as a height that fell 2 standard deviations below the mean height of the peer population, without any identifiable aetiology [26]. Atopic cough was defined as a nonproductive cough with an atopic tendency and cough hypersensitivity but without nonspecific bronchial hyperresponsiveness. Thyroid disorders were mainly referred to as hyperthyroidism and hypothyroidism in this study.

The distributions of C3-epi-25(OH)D3 levels and %C3-epi-25(OH)D3 for these 20 common paediatric diseases are described in terms of the median (*P*_25_, *P*_75_) [*Min*, *Max*] (Table 1). With age, sex, season, and 25(OH)D3 levels as confounding factors, we performed pairwise comparisons via post hoc covariance analysis. Compared with those in healthy controls, C3-epi-25(OH)D3 levels were lower only in patients with atopic cough (*t*=-3.925, *Padj* = 0.037) and short stature (*t*=-4.094, *Padj* = 0.029). %C3-epi-25(OH)D3 decreased in patients with atopic cough (*t*=-3.753, *Padj* = 0.046), short stature (*t*=-4.242, *Padj* = 0.024), hypovitaminosis D (*t*=-4.152, *Padj* = 0.027), trachitis/bronchitis (*t*=-3.827, *Padj* = 0.042), and attention-deficit/hyperactivity disorder (*t*=-3.719, *Padj* = 0.048), whereas it increased in patients with rickets (*t* = 4.199, *Padj* = 0.026), nephrotic syndrome (*t* = 6.795, *Padj* < 0.001), and lymphocytic leukaemia (*t* = 3.919, *Padj* = 0.037). This finding showed that %C3-epi-25(OH)D3 could reveal the change in C3-epi-25(OH)D3 generation under those disease conditions more clearly than its levels.

**Table 1:** The C3-epi-25(OH)D3 levels and percentages in 20 pediatric common diseases {*Median* (*P*_25_, *P*_75_) [*min*, *max*]}, and their difference significances after adjusting 25(OH)D3 level especially

Disease	*n*	25(OH)D3 (ng/mL)	C3-epi-25(OH)D3 (ng/mL)	%C3-epi-25(OH)D3 (%)
Healthy control	805	27.08 (22.42, 32.64) [9.23, 54.66]	1.59 (1.08, 2.31) [0.10, 7.05]	5.71 (4.00, 7.50) [0.39, 15.61]
Hypovitaminosis D	196	14.98 (11.89, 17.60) [5.65, 19.35]	0.79 (0.54, 1.06) [0.12, 2.52]	**5.25 (3.82**,** 6.99) [1.05**, 11.97] *
Short stature	623	30.05 (25.48, 34.44) [16.00, 54.48]	1.68 (1.22, 2.34) [0.28, 7.79]	5.39 (4.05, 7.04) [0.87, 15.99]
Rickets	83	15.33 (12.61, 17.98) [6.26, 19.65]	0.94 (0.58, 1.27) [0.19, 3.30]	**5.93 (4.44**,** 7.57) [1.40**, 15.62] *
Growing arthralgia	46	19.22 (14.43, 28.43) [6.48, 45.98]	1.01 (0.64, 1.54) [0.17, 3.20]	4.91 (3.30, 7.58) [1.59, 10.42]
Pneumonia	44	25.70 (21.27, 34.65) [8.32, 53.44]	1.43 (1.06, 2.04) [0.35, 4.84]	5.09 (4.18, 6.72) [2.48, 11.95]
Atopic cough	170	25.41 (20.28, 31.23) [9.95, 50.05]	**1.42 (0.89**,** 2.14) [0.19**, 4.85] *	**5.41 (3.66**,** 7.34) [0.72**, 14.07] *
Trachitis/ bronchitis	126	24.54 (19.11, 31.60) [4.03, 53.19]	1.32 (0.91, 1.92) [0.11, 6.21]	**5.13 (4.08**,** 6.93) [0.64**, 13.55] *
Upper respiratory infection	190	25.46 (19.13, 32.58) [8.32, 53.93]	1.43 (0.95, 2.25) [0.26, 7.71]	5.71 (3.99, 7.50) [0.99, 14.73]
Cough variant asthma	401	27.65 (21.64, 35.01) [6.64, 54.71]	1.67 (1.08, 2.59) [0.22, 7.45]	5.89 (4.10, 8.06) [0.96, 15.81]
Nephrotic syndrome	69	17.49 (7.97, 28.77) [0.95, 40.77]	1.10 (0.62, 2.15) [0.12, 7.21]	**6.89 (5.01**,** 9.45) [1.44**, 20.25] *
Thyroid disorder	26	23.22 (16.50, 30.45) [5.84, 46.18]	1.32 (0.74, 1.92) [0.28, 4.12]	5.21 (3.45, 7.32) [0.87, 14.82]
Growth hormone deficiency	38	25.67 (22.23, 29.97) [13.84, 44.52]	1.30 (1.03, 1.72) [0.38, 4.03]	5.14 (4.04, 6.65) [1.66, 10.92]
Sexual precocity	329	24.38 (19.94, 29.79) [6.77, 50.96]	1.23 (0.78, 1.74) [0.15, 5.68]	4.91 (3.24, 6.87) [0.45, 15.41]
Tic distic disorder	218	24.04 (18.80, 28.86) [7.69, 44.64]	1.31 (0.85, 1.92) [0.21, 4.88]	5.57 (4.07, 7.71) [0.69, 15.07]
Epilepsy	121	24.49 (19.03, 32.07) [5.70, 50.42]	1.30 (0.87, 1.95) [0.30, 9.42]	5.18 (3.90, 7.16) [1.41, 17.22]
Attention-deficit/ hyperactivity disorder	107	23.19 (18.55, 28.45) [7.39, 45.62]	1.28 (0.88, 1.73) [0.22, 3.68]	**5.61 (3.68**,** 6.70) [0.97**, 11.71] *
Mental retardation/ developmental disabilities	54	30.56 (24.78, 36.62) [15.68, 52.70]	2.31 (1.43, 3.16) [0.18, 4.81]	6.20 (5.02, 9.33) [0.54, 15.40]
Sleep disorders	66	23.20 (19.74, 28.61) [8.24, 44.85]	1.23 (0.87, 1.69) [0.20, 4.29]	4.72 (3.72, 6.97) [1.68, 12.14]
Paediatric malnutrition	29	25.41 (18.43, 34.95) [6.94, 44.85]	**1.11 (0.62**,** 1.72) [0.18**, 3.81] *	**4.65 (3.33**,** 5.83) [0.80**, 9.96] *
Lymphocytic leukemia	26	28.30 (21.87, 30.95) [7.48, 41.84]	1.87 (1.57, 2.18) [0.67, 4.23]	**6.88 (5.29**,** 7.73) [4.25**, 11.74] *

*Note* * Compared to healthy controls with Bonferroni correction, *Padj* < 0.05. 25(OH)D3 levels were only exhibited and not compared. After adjusted for gender, age, season, and 25(OH)D3 level, C3-epi-25(OH)D3 levels changed significantly in only two diseases, compared with eight diseases for %C3-epi-25(OH)D3. In Rickets and Nephrotic syndrome, a separation phenomenon of decreased C3-epi-25(OH)D3 level due to tracking 25(OH)D3 level and increased %C3-epi-25(OH)D3 were observed, suggesting that %C3-epi-25(OH)D3 might have an ability to reflect the pathological generation of C3-epi-25(OH)D3

### Bivariate relationship analysis among C3-epi-25(OH)D3 levels, %C3-epi-25(OH)D3, and 25(OH)D3 levels

**Fig. 3:**
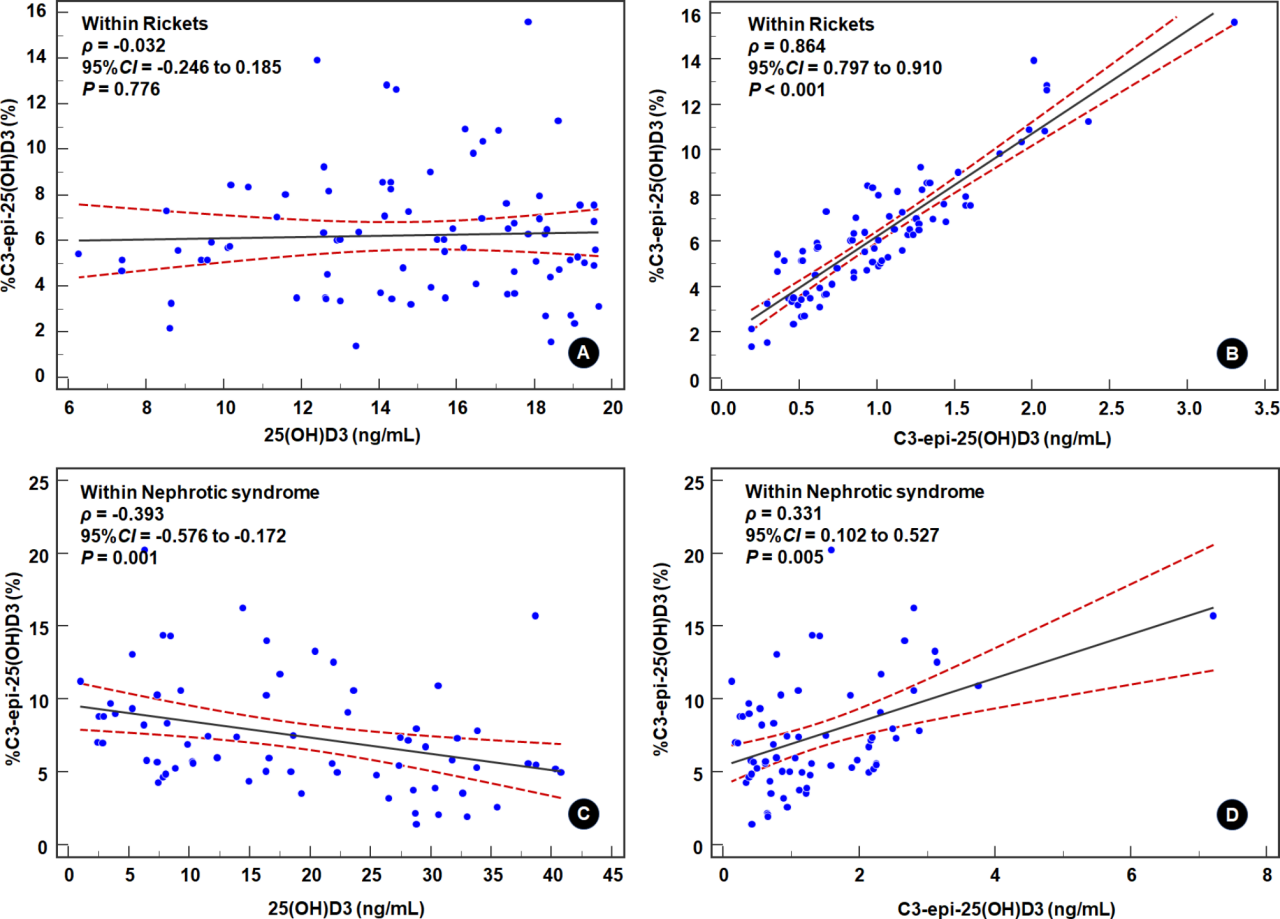
Changes in the correlation of %C3-epi-25(OH)D3 with 25(OH)D3 and C3-epi-25(OH)D3 levels in patients with rickets or nephrotic syndrome. *Note* The significance level adopted the Bonferroni correction, with α = 0.05/3 ≈ 0.017. The black solid line represents the correlation coefficient, and the red dotted line delineates the 95% confidence interval for the correlation coefficient. The correlations of %C3-epi-25(OH)D3 with 25(OH)D3 and C3-epi-25(OH)D3 levels are distinctly different in patients with nephrotic syndrome than in healthy controls and those with other diseases (for example, Rickets). The correlation between %C3-epi-25(OH)D3 and 25(OH)D3 changed from a noncorrelated correlation to a negative correlation, and the correlation between %C3-epi-25(OH)D3 and C3-epi-25(OH)D3 weakened

We observed Spearman bivariate correlations between 25(OH)D3, C3-epi-25(OH)D3, and %C3-epi-25(OH)D3. Moreover, significance was determined by employing a Bonferroni-corrected significance level of α = 0.05/3 ≈ 0.017. The results are shown in Table 2. Under healthy and disease conditions, C3-epi-25(OH)D3 levels were positively correlated with 25(OH)D3 levels (*ρ* = 0.318–0.678, all *P* < 0.017). Moreover, %C3-epi-25(OH)D3 was positively correlated with C3-epi-25(OH)D3 levels (*ρ* = 0.637–0.880, all *P* < 0.001) but was not correlated with 25(OH)D3 levels (*ρ*=-0.219–0.162, all *P* > 0.017), except for those associated with nephrotic syndrome (shown in Fig. 3: vs. C3-epi-25(OH)D3: *ρ* = 0.331, *P* = 0.005; vs. 25(OH)D3: *ρ*=-0.393, *P* = 0.001). These results suggested that the high traceability of C3-epi-25(OH)D3 levels to 25(OH)D3 levels was always present in healthy and diseased conditions. Thus, the use of C3-epi-25(OH)D3 levels to explore the relationship between C3-epi-25(OH)D3 and diseases may yield inappropriate results. However, the use of %C3-epi-25(OH)D3 may curb this problem because %C3-epi-25(OH)D3 demonstrated a high correlation with C3-epi-25(OH)D3 levels but not with 25(OH)D3 levels under most conditions.

**Table 2:** Spearman bivariate correlations between 25(OH)D3, C3-epi-25(OH)D3, and %C3-epi-25(OH)D3 {*ρ*(95%*CI*), *P*}

Disease	*n*	C3-epi-25(OH)D3 vs. 25(OH)D3	%C3-epi-25(OH)D3 vs. 25(OH)D3	%C3-epi-25(OH)D3 vs. C3-epi-25(OH)D3
Healthy control	805	**0.441 (0.384 to 0.495)**,** < 0.001**	-0.025 (-0.094 to 0.045), 0.485	**0.863 (0.844 to 0.879)**,** < 0.001**
Hypovitaminosis D	196	**0.496 (0.382 to 0.595)**,** < 0.001**	0.022 (-0.118 to 0.162), 0.759	**0.842 (0.795 to 0.878)**,** < 0.001**
Short stature	623	**0.330 (0.258 to 0.398)**,** < 0.001**	-0.113 (-0.190 to 0.035), 0.271	**0.880 (0.860 to 0.896)**,** < 0.001**
Rickets	83	**0.432 (0.238 to 0.592)**,** < 0.001**	-0.032 (-0.246 to 0.185), 0.776	**0.864 (0.797 to 0.910)**,** < 0.001**
Growing arthralgia	46	**0.635 (0.423 to 0.781)**,** < 0.001**	-0.053 (-0.338 to 0.241), 0.726	**0.689 (0.499 to 0.816)**,** < 0.001**
Pneumonia	44	**0.571 (0.330 to 0.742)**,** < 0.001**	-0.063 (-0.354 to 0.238), 0.682	**0.727 (0.548 to 0.842)**,** < 0.001**
Atopic cough	170	**0.638 (0.540 to 0.720)**,** < 0.001**	0.162 (0.011 to 0.305), 0.035	**0.848 (0.800 to 0.886)**,** < 0.001**
Trachitis/ bronchitis	126	**0.678 (0.571 to 0.763)**,** < 0.001**	0.044 (-0.132 to 0.217), 0.624	**0.717 (0.620 to 0.793)**,** < 0.001**
Upper respiratory infection	190	**0.666 (0.579 to 0.738)**,** < 0.001**	0.111 (-0.032 to 0.249), 0.128	**0.788 (0.727 to 0.836)**,** < 0.001**
Cough variant asthma	401	**0.592 (0.524 to 0.652)**,** < 0.001**	0.035 (-0.0633 to 0.132), 0.486	**0.800 (0.762 to 0.833)**,** < 0.001**
Nephrotic syndrome	69	**0.672 (0.517 to 0.784)**,** < 0.001**	**-0.393 (-0.576 to -0.172)**,** 0.001**	**0.331 (0.102 to 0.527)**,** 0.005**
Thyroid disorder	26	**0.468 (0.099 to 0.724)**,** 0.016**	-0.046 (-0.425 to 0.348), 0.825	**0.817 (0.628 to 0.915)**,** < 0.001**
Growth hormone deficiency	38	**0.464 (0.170 to 0.683)**,** 0.003**	-0.134 (-0.435 to 0.194), 0.423	**0.774 (0.604 to 0.877)**,** < 0.001**
Sexual precocity	329	**0.344 (0.245 to 0.436)**,** < 0.001**	-0.166 (-0.270 to -0.059), 0.025	**0.834 (0.798 to 0.864)**,** < 0.001**
Tic distic disorder	218	**0.484 (0.376 to 0.580)**,** < 0.001**	-0.066 (-0.197 to 0.068), 0.336	**0.805 (0.753 to 0.847)**,** < 0.001**
Epilepsy	121	**0.588 (0.458 to 0.694)**,** < 0.001**	-0.046 (-0.222 to 0.134), 0.619	**0.727 (0.630 to 0.801)**,** < 0.001**
Attention-deficit/ hyperactivity disorder	107	**0.543 (0.393 to 0.664)**,** < 0.001**	0.003 (-0.187 to 0.193), 0.973	**0.795 (0.712 to 0.855)**,** < 0.001**
Mental retardation/ developmental disabilities	54	**0.367 (0.111 to 0.578)**,** 0.006**	-0.170 (-0.419 to 0.102), 0.218	**0.802 (0.680 to 0.880)**,** < 0.001**
Sleep disorders	66	**0.318 (0.082 to 0.520)**,** 0.009**	-0.185 (-0.409 to 0.0598), 0.137	**0.834 (0.742 to 0.895)**,** < 0.001**
Paediatric malnutrition	29	**0.629 (0.342 to 0.809)**,** < 0.001**	-0.025 (-0.388 to 0.345), 0.898	**0.701 (0.450 to 0.849)**,** < 0.001**
Lymphocytic leukemia	26	**0.566 (0.228 to 0.782)**,** 0.003**	-0.219 (-0.559 to 0.184), 0.283	**0.637 (0.489 to 0.865)**,** < 0.001**

*Note* The significant level adopted Bonferroni correction as α = 0.05/3 ≈ 0.017. Within health and disease conditions, C3-epi-25(OH)D3 levels positively correlated to 25(OH)D3 levels, indirectly confirming that the peculiarity of C3-epi-25(OH)D3 highly tracking 25(OH)D3 was universal; %C3-epi-25(OH)D3 strongly and positively correlated to C3-epi-25(OH)D3 levels and not correlated to 25(OH)D3 levels except for in nephrotic syndrome, indicating that %C3-epi-25(OH)D3 could reflect C3-epi-25(OH)D3 levels and was independent of 25(OH)D3 levels

### Degree of the relationship of C3-epi-25(OH)D3 levels and %C3-epi-25(OH)D3 with the occurrence of various diseases

Multivariate logistic regression was used to analyse the degree of the correlation of C3-epi-25(OH)D3 or %C3-epi-25(OH)D3 with various diseases, with age, sex, season, and 25(OH)D3 levels as confounding factors. The same analysis with only age, sex, and season as confounding factors was also performed to compare the differences with and without correction for 25(OH)D3 levels. The results showed that before and after adjusting for 25(OH)D3, the degree of correlation of C3-epi-25(OH)D3 with multiple diseases differed significantly (Table 3); however, that of %C3-epi-25(OH)D3 with various diseases was very similar (Table 4). Based on our adjusted results, C3-epi-25(OH)D3 was only associated with the occurrence of four diseases: short stature (*OR* = 1.32, *P* < 0.001), nephrotic syndrome (*OR* = 1.61, *P* = 0.012), lymphocytic leukaemia (*OR* = 1.71, *P* = 0.010), and paediatric malnutrition (*OR* = 0.51, *P* = 0.031). However, %C3-epi-25(OH)D3 was associated with the occurrence of six diseases: short stature (*OR* = 1.10, *P* < 0.001), nephrotic syndrome (*OR* = 1.21, *P* < 0.001), lymphocytic leukaemia (*OR* = 1.21, *P* = 0.007), paediatric malnutrition (*OR* = 0.80, *P* = 0.017), rickets (*OR* = 1.19, *P* = 0.012), and hypovitaminosis D (*OR* = 0.92, *P* = 0.040). These results suggested that %C3-epi-25(OH)D3 was more suitable than C3-epi-25(OH)D3 for exploring the correlation between C3-epi-25(OH)D3 generation and disease occurrence.

**Table 3:** The relation of C3-epi-25(OH)D3 levels with 20 pediatric common diseases

Disease	Age, gender, and season as the confounding factors			Age, gender, season, and 25(OH)D3 as the confounding factors		
	OR (95% CI)	Wald	*P*	OR (95% CI)	Wald	*P*
Hypovitaminosis D	**0.19 (0.14 to 0.27)**	**85.876**	**< 0.001**	0.74 (0.51 to 1.08)	2.461	0.117
Short stature	**1.67 (1.49 to 1.88)**	**72.986**	**< 0.001**	**1.32 (1.16 to 1.50)**	**17.088**	**< 0.001**
Rickets	**0.38 (0.24 to 0.58)**	**19.675**	**< 0.001**	1.42 (0.90 to 2.24)	2.214	0.137
Growing arthralgia	**0.59 (0.36 to 0.95)**	**4.745**	**0.029**	0.81 (0.48 to 1.37)	0.596	0.440
Pneumonia	**0.70 (0.49 to 0.99)**	**4.034**	**0.045**	0.80 (0.54 to 1.18)	1.260	0.262
Atopic cough	**0.74 (0.61 to 0.90)**	**8.683**	**0.003**	0.88 (0.70 to 1.10)	1.303	0.254
Trachitis/ bronchitis	**0.72 (0.57 to 0.91)**	**7.725**	**0.005**	0.84 (0.65 to 1.08)	1.809	0.179
Upper respiratory infection	0.90 (0.76 to 1.08)	1.253	0.263	1.07 (0.88 to 1.30)	0.478	0.490
Cough variant asthma	1.03 (0.91 to 1.17)	0.273	0.601	1.11 (0.96 to 1.27)	2.104	0.147
Nephrotic syndrome	0.72 (0.51 to 1.01)	3.701	0.054	**1.61 (1.11 to 2.34)**	**6.311**	**0.012**
Thyroid disorder	1.09 (0.66 to 1.80)	0.127	0.722	1.16 (0.67 to 2.01)	0.266	0.606
Growth hormone deficiency	0.86 (0.57 to 1.31)	0.501	0.479	0.84 (0.54 to 1.32)	0.558	0.455
Sexual precocity	1.02 (0.85 to 1.22)	0.038	0.845	1.01 (0.83 to 1.23)	0.008	0.928
Tic distic disorder	0.87 (0.72 to 1.05)	2.228	0.136	1.04 (0.85 to 1.28)	0.144	0.705
Epilepsy	**0.79 (0.63 to 1.00)**	**3.970**	**0.046**	0.88 (0.68 to 1.13)	1.023	0.312
Attention-deficit/ hyperactivity disorder	**0.72 (0.54 to 0.96)**	**5.116**	**0.024**	0.86 (0.63 to 1.17)	0.935	0.333
Mental retardation/ developmental disabilities	0.94 (0.73 to 1.22)	0.228	0.633	1.00 (0.75 to 1.34)	0.000	0.992
Sleep disorders	**0.68 (0.48 to 0.97)**	**4.528**	**0.033**	0.77 (0.52 to 1.13)	1.795	0.180
Paediatric malnutrition	**0.48 (0.27 to 0.85)**	**6.427**	**0.011**	**0.51 (0.28 to 0.94)**	**4.649**	**0.031**
Lymphocytic leukemia	**1.66 (1.15 to 2.39)**	**7.336**	**0.007**	**1.71 (1.13 to 2.58)**	**6.554**	**0.010**

*Note* The multivariate logistic regression analysis was used with healthy controls as reference. The relation assessment about C3-epi-25(OH)D3 levels with 20 pediatric common diseases after adjusting 25(OH)D3 was significantly differed from before adjusting 25(OH)D3

**Table 4:** The relation of %C3-epi-25(OH)D3 with 20 pediatric common diseases

Disease	Age, gender, and season as the confounding factors			Age, gender, season, and 25(OH)D3 as the confounding factors		
	OR (95% CI)	Wald	*P*	OR (95% CI)	Wald	*P*
Hypovitaminosis D	1.02 (0.95 to 1.09)	0.344	0.558	**0.92 (0.86 to 1.00)**	**4.236**	**0.040**
Short stature	**1.06 (1.01 to 1.10)**	**5.874**	**0.015**	**1.10 (1.05 to 1.15)**	**16.219**	**< 0.001**
Rickets	**1.15 (1.05 to 1.25)**	**9.844**	**0.002**	**1.19 (1.08 to 1.29)**	**6.926**	**0.012**
Growing arthralgia	1.00 (0.88 to 1.13)	0.000	0.984	0.96 (0.84 to 1.09)	0.362	0.547
Pneumonia	0.95 (0.83 to 1.07)	0.749	0.387	0.94 (0.82 to 1.06)	1.061	0.303
Atopic cough	0.95 (0.89 to 1.02)	1.700	0.192	0.94 (0.88 to 1.01)	2.749	0.097
Trachitis/ bronchitis	0.95 (0.88 to 1.03)	1.634	0.201	0.93 (0.86 to 1.01)	2.789	0.095
Upper respiratory infection	1.02 (0.95 to 1.08)	0.241	0.624	1.00 (0.94 to 1.07)	0.006	0.938
Cough variant asthma	1.03 (0.98 to 1.08)	1.567	0.211	1.03 (0.98 to 1.08)	1.318	0.251
Nephrotic syndrome	**1.28 (1.17 to 1.39)**	**33.087**	**< 0.001**	**1.21 (1.11 to 1.32)**	**18.095**	**< 0.001**
Thyroid disorder	1.05 (0.90 to 1.23)	0.387	0.534	1.04 (0.89 to 1.22)	0.269	0.604
Growth hormone deficiency	0.95 (0.83 to 1.09)	0.468	0.494	0.95 (0.83 to 1.09)	0.453	0.501
Sexual precocity	1.01 (0.95 to 1.07)	0.110	0.740	1.01 (0.95 to 1.07)	0.163	0.687
Tic distic disorder	1.03 (0.97 to 1.10)	1.090	0.296	1.02 (0.95 to 1.08)	0.236	0.627
Epilepsy	0.96 (0.89 to 1.04)	0.962	0.327	0.95 (0.88 to 1.03)	1.570	0.210
Attention-deficit/ hyperactivity disorder	0.97 (0.89 to 1.06)	0.497	0.481	0.94 (0.86 to 1.03)	1.561	0.211
Mental retardation/ developmental disabilities	1.02 (0.92 to 1.14)	0.198	0.656	1.02 (0.92 to 1.14)	0.134	0.714
Sleep disorders	0.95 (0.86 to 1.06)	0.715	0.398	0.94 (0.84 to 1.05)	1.344	0.246
Paediatric malnutrition	**0.82 (0.69 to 0.98)**	**4.584**	**0.032**	**0.80 (0.66 to 0.96)**	**5.672**	**0.017**
Lymphocytic leukemia	**1.20 (1.05 to 1.38)**	**6.947**	**0.008**	**1.21 (1.06 to 1.40)**	**7.402**	**0.007**

*Note* The multivariate logistic regression analysis was used with healthy controls as reference. The relation assessment about C3-epi-25(OH)D3 levels with 20 pediatric common diseases was almost perfectly consistent both before and after adjusting 25(OH)D3

## Discussion

C3-epi-25(OH)D has been used as a designated indicator in nationally representative surveys in a few developed countries, such as the National Health and Nutrition Examination Survey [15, 16]. However, little is known regarding the pathological importance of C3-epi-25(OH)D, except for the overestimation of vitamin D storage [27–29]. In this study, we demonstrated the importance of correcting the properties of C3-epi-25(OH)D3, which can sensitively track 25(OH)D3. We believe that with a simple correction (which is a calculation of %C3-epi-25(OH)D3), a relatively stable degree of relationship between C3-epi-25(OH)D3 generation and the occurrence of multiple paediatric diseases can be obtained. This finding suggests that the C3-epi-25(OH)D3%, not its level, may be a potentially better biomarker for investigating the pathological relevance of C3-epi-25(OH)D3 generation.

Prior to our disease investigations, we observed differences regarding sex, age, and season in C3-epi-25(OH)D3 levels and %C3-epi-25(OH)D3 in healthy underage controls. After correcting for the other two influencing factors, we obtained the same conclusion for the C3-epi-25(OH)D3 levels and %C3-epi-25(OH)D3. Both values were greater in the infant period than in the other periods, as well as in spring and summer than in autumn and winter. For C3-epi-25(OH)D3 levels, the age difference was consistent with previous reports, and the seasonal difference was similar to that reported in other studies [18, 30]. The highest level in the summer is attributed mainly to an increase in 25(OH)D3 generation due to sunlight [31, 32], which may increase C3-epi-25(OH)D3 generation according to the properties of C3-epi-25(OH)D3, which is a sensitive tracker of 25(OH)D3 [20]. However, the differences in %C3-epi-25(OH)D3 based on age and season imply that C3-epi-25(OH)D3 generation may be physiologically increased during infancy, spring, and summer. The specific reasons and mechanisms underlying this phenomenon require further clarification. In addition, some studies have reported sex differences and the reverse changes during spring and winter [33, 34]. Our results suggest the opposite, possibly due to the influence of ethnic, regional, population, and interlaboratory differences [35, 36]. We do not consider the influence of sunscreens on the underage population [37] because sunscreens are rarely used in this population. Currently, standardized measurement methods and reference materials for C3-epi-25(OH)D are lacking. To better assess 25(OH)D metabolites and their percentages in various regions and countries, it is necessary to formulate a 25(OH)D standardization measurement programme and develop a standard reference material for C3-epi-25(OH)D.

The physiological changes in C3-epi-25(OH)D3 levels and %C3-epi-25(OH)D3 suggest that the effects of age, season, and sex should be considered in relevant studies, similar to other vitamin D metabolite studies. However, even after adjusting for sex, age, and season, the relationship between C3-epi-25(OH)D3 and disease occurrence remains controversial [38–40]. Further consideration revealed that the ability of C3-epi-25(OH)D3 to track 25(OH)D3 effectively could not be ignored. Our results revealed a strong correlation between C3-epi-25(OH)D3 and 25(OH)D3 under healthy and diseased conditions, indirectly confirming the prevalence of this property. Owing to the complexities of C3-epi-25(OH)D in terms of its properties and influencing factors [19, 41], its contribution to disease management remains unclear. However, the use of C3-epi-25(OH)D to assess disease conditions requires more reliable methods.

Surprisingly, with a simple correction involving calculating the %C3-epi-25(OH)D, the relationship between C3-epi-25(OH)D3 generation and disease occurrence was better understood. Our results revealed that, after adjusting for influencing factors, including 25(OH)D3 levels, among 20 common paediatric diseases, C3-epi-25(OH)D3 levels changed significantly only in two diseases, whereas %C3-epi-25(OH)D changed in eight diseases. This result suggested that %C3-epi-25(OH)D3 could better reflect the pathological relevance of C3-epi-25(OH)D3 generation, which will be helpful for further research on other relevant details. Subsequent analysis revealed that the C3-epi-25(OH)D3 level was notably different, whereas the %C3-epi-25(OH)D3 level was almost consistent in terms of its relationship with disease occurrence before and after adjusting for 25(OH)D3 levels. Therefore, %C3-epi-25(OH)D3 may be more suitable than the C3-epi-25(OH)D3 level for exploring the correlation between C3-epi-25(OH)D3 generation and disease occurrence.

Our correlation results regarding %C3-epi-25(OH)D3 with disease occurrence are consistent with the pathological conditions of vitamin D metabolism during the course of disease occurrence and development. Among the 20 common paediatric diseases, we discovered a significant correlation between %C3-epi-25(OH)D3 and six of these diseases. Specifically, it was positively correlated with short stature, nephrotic syndrome, lymphocytic leukaemia, and rickets but negatively correlated with paediatric malnutrition and hypovitaminosis D. These diseases involve the metabolism and function of vitamin D, including its absorption, synthesis, physiological utilization, and immune regulation. Therefore, there was a positive correlation between increased %C3-epi-25(OH)D3 and the aforementioned four diseases. An increase in %C3-epi-25(OH)D3 indicates a relative increase in C3-epimer levels or a relative decrease in 25(OH)D3 levels. In individuals with high %C3-epi-25(OH)D3, if the sum of C3-epimer and non-C3 epimer levels remains constant, then it may exacerbate 25(OH)D deficiency. These findings may provide a rationale for the pathological risks associated with elevated levels of %C3-epi-25(OH)D3. Moreover, the negative correlation between %C3-epi-25(OH)D3 and paediatric malnutrition as well as hypovitaminosis D is easily understood. An increase in %C3-epi-25(OH)D3 indicates an increase in C3-epi-25(OH)D3 levels, which also implies an increase in 25(OH)D3 levels due to the high tracking ability of C3-epi-25(OH)D3 with 25(OH)D3. From this point, C3-epi-25(OH)D3 or %C3-epi-25(OH)D3 is more like a follower of 25(OH)D3 and demonstrates a negative correlation with both paediatric malnutrition and hypovitaminosis D. Thus, to correctly understand the relationship between %C3-epi-25(OH)D3 and disease occurrence, it is necessary to consider not only the influence of the metabolic changes in C3-epi-25(OH)D3 itself but also its ability to effectively track 25(OH)D3 levels. Furthermore, our findings suggest that the correlations between %C3-epi-25(OH)D and the levels of 25(OH)D3 and C3-epi-25(OH)D3 in patients with nephrotic syndrome differ significantly from those reported in healthy controls and from those with other diseases. These distinctions may be attributed to the crucial role of the kidneys in vitamin D metabolism, as any renal impairment can lead to alterations in the intrarenal enzymes responsible for vitamin D metabolism [13, 42].

Vitamin D metabolism genetic polymorphism is implicated in various pathways of vitamin D, and 25(OH)D3 epimerization process is also influenced by the interplay of genetic factors and various internal environmental factors related to vitamin D metabolism [22, 23]. Due to highly tracking to 25(OH)D levels [20], the generation of C3-epi-25(OH)D3 is also influenced by these factors. An investigation of single nucleotide polymorphisms in C3-epi-25(OH)D confirmed that the genetic determinants and potential factors of C3-epimers differ from those of non-C3-epimers [43]. These findings suggest that the pathological generation of C3-25(OH)D3 objectively occurs in vivo. Overall, our study may help resolve the controversy regarding the pathological relevance of C3-epi-25(OH)D3 generation. We hypothesize that the unique tracking ability of C3-epi-25(OH)D3 for 25(OH)D3 conceals and even eliminates exposure to its pathological generation in vivo. Therefore, in relevant research regarding C3-epi-25(OH)D3, correction should be made for the effect of 25(OH)D3, in addition to the effects of sex, age, and season. In contrast with the C3-epi-25(OH)D3 level, the %C3-epi-25(OH)D3 level can circumvent the influence of the 25(OH)D3 level and may be conducive to accurately exploring the pathological significance of 25(OH)D3 epimerization. Therefore, further studies on the causality between diseases and %C3-epi-25(OH)D3, as well as genetic studies on these diseases, which are influenced by a higher %C3-epi-25(OH)D3, are necessary. Moreover, calculating %C3-epi-25(OH)D3 may be a relatively simple and effective correction method; however, its pathological relevance requires further exploration in many in-depth studies. Currently, IOM and EnS/JES recommend the use of mass spectrometry for 25(OH)D3 analysis [1, 2], while the determination of C3-epi-25(OH)D3 is still in its nascent stages. However, neither of these methods are performed routinely. Owing to the advantages of 25(OH)D3 in terms of biological activity, origin, synthesis, and nutritional assessment, as well as its obvious effect on vitamin D storage and possible pathological value of high C3-epi-25(OH)D3 content, further exploration of both could significantly contribute to advancements in pathology and nutrition.

This study had a few limitations. The most prominent limitation was that it was only a preliminary and crude relationship analysis for %C3-epi-25(OH)D3 with multiple diseases; therefore, further detailed research is needed for each specific disease. Second, because of the different types of diseases, it was impossible to match the data regarding age, sex, and season from patients with each disease to healthy controls; however, our results were adjusted for age, sex, and season. Third, this was a single-centre, cross-sectional study; therefore, causal associations, racial differences, and regional differences cannot be determined. In addition, other potentially influential factors, such as dietary habits, outdoor activity, and sunscreen use, were not considered.

## Conclusions

To the best of our knowledge, this is the first study to investigate the differential performance of C3-epi-25(OH)D3 levels and %C3-epi-25(OH)D3 in various common paediatric diseases. Our results emphasize that the C3-epi-25(OH)D percentage, not the C3-epi-25(OH)D level, may be a potential biomarker for better interpretation of the relationship between C3-epi-25(OH)D3 and multiple diseases. The %C3-epi-25(OH)D3 can correct the concern regarding the C3-epi-25(OH)D3 level, highly tracking the 25(OH)D3 level. Moreover, %C3-epi-25(OH)D3 can reflect the physiological generation and pathological increase in the 25(OH)D3 C3-epimer. This simple calculation method is expected to pave the way for correctly exploring the details of C3-epi-25(OH)D generation, further promoting an in-depth study of its pathological relevance.

## Data Availability

All the data generated or analysed during this study are included in this published article and its supplementary information files.

## Abbreviations

%C3: Epi-25(OH)D3
percentage of C3: Epimer of 25-hydroxyvitamin D
25(OH)D: 25-hydroxyvitamin D
APCI: Atmospheric pressure chemical ionization
C3-epi-25(OH)D: C3-epimer of 25-hydroxyvitamin D
CI: Confidence interval
EnS: Endocrine Society
IOM: Institute of Medicine
JES: Japan Endocrine Society
OR: Odds ratio
